# Does the classroom context moderate the effects of internalizing problems and peer status on peer victimization? Testing a vulnerability-by-context model

**DOI:** 10.1177/01650254251333684

**Published:** 2025-05-16

**Authors:** Claire F. Garandeau, Sarah T. Malamut, Lydia Laninga-Wijnen, Christina Salmivalli

**Affiliations:** University of Turku, Finland

**Keywords:** Bullying, victimization, anxiety, depression, peer status, classroom context, multilevel modeling

## Abstract

This two-wave study investigated whether the concurrent and prospective effects of internalizing symptoms and peer status (being liked/disliked) on peer-reported and self-reported victimization were moderated by the classroom prevalence of internalizing symptoms and peer status, respectively, and by classroom size. Multilevel regression analyses were conducted with data collected from 2,641 Finnish secondary school students (*M*_age_ = 13.71; 48.8% girls) at the beginning (September 2022) and in the middle of the school year (January 2023). Concurrently, higher levels of internalizing problems and lower status were associated with higher peer-reported and self-reported victimization, but these associations were not moderated by the classroom prevalence of these individual characteristics nor by classroom size. Longitudinally, higher levels of internalizing problems and lower status predicted more self-reported victimization 5 months later, but only lower status predicted higher peer-reported victimization over time. No moderating effect of the classroom features of interest were found.

In late childhood and early adolescence, individuals increasingly depend on peers to fulfill their need for acceptance and intimacy (Sullivan, 1953) and the desire for popularity among peers is prioritized (LaFontana & Cillessen, 2010). Thus, becoming the target of school bullying, which is intentional aggression repeated against a peer in a more vulnerable position, can be a serious threat to youth’s healthy social and emotional development. Indeed, victimization by peers leads to both mental health difficulties, such as depression and anxiety (Christina et al., 2021) and social adjustment problems, such as peer rejection (Demol et al., 2020), lower peer acceptance (Sentse et al., 2015) and lack of friend support (Siennick & Turanovic, 2024). Understanding the conditions that increase the risks that a student becomes the target of bullying is therefore essential. In a recent global survey, 30.5% of the 12-to-17-year-old respondents (8.4% in Europe) reported being bullied by their school peers at least once in the past month (Biswas et al., 2020), suggesting that school victimization is highly prevalent. However, some youth are at higher risk than others. Two characteristics have emerged as key risk factors for victimization in the literature: internalizing problems (e.g., Christina et al., 2021) and low peer status (e.g., being disliked; Demol et al., 2020). In this study, we use the term *peer status* to refer to the extent to which adolescents are liked or disliked by peers.^1^

Bullying is often initiated within a larger social context and the classroom environment is particularly relevant as most peer interactions occur among classmates. Therefore, the extent to which psychological or social problems predict victimization may depend on characteristics of the classroom context, as suggested in some cross-sectional studies (e.g., Kärnä et al., 2010) and a few longitudinal studies (Guimond et al., 2018; Kaufman et al., 2022; Serdiouk et al., 2015). In line with the person-group dissimilarity model (Wright et al., 1986), which postulates that individuals are more likely to be rejected by the group when they deviate from the group norm, we expect that internalizing problems and low peer status will make students even more vulnerable to victimization when these characteristics are less common among classmates. Moreover, the concurrent associations of social anxiety and peer rejection with peer victimization were found to be stronger in smaller classrooms (Saarento et al., 2013), suggesting that restricted peer networks may exacerbate the effects of these vulnerability factors on victimization.

It is known that bullying is driven both by group dynamics and by the personal characteristics of those involved, but more specific information on how they interact is essential for more effective anti-bullying guidelines (e.g., beyond identifying vulnerable students, teachers should know which social contexts can put these students especially at risk for victimization). To better understand whether internalizing problems and low peer status are stronger risk factors for victimization in certain contexts, more studies using longitudinal, multilevel designs are needed. To this end, this study puts to the test a *vulnerability-by-context model for peer victimization* in a sample of early adolescents by investigating the moderating effects of two classroom-level factors on the effects of internalizing symptoms and peer status at the beginning of the school year on concurrent and later victimization (5 months later), namely, the classroom prevalence of internalizing symptoms and peer status, and classroom size.

## Vulnerability Factors for Victimization

Ample research shows that being anxious and depressed puts youth at risk for peer victimization (see Christina et al., 2021). Presumably, these psychological difficulties work as signals of vulnerability, which makes aggressors less likely to face backlash and the bullying more rewarding for them. According to the interpersonal theory of depression (Coyne, 1976) and the social risk hypothesis (Allen & Badcock, 2003), depressed individuals may withdraw from social situations and display submissiveness to avoid confrontations. When faced with bullying, anxious and depressed youth may be less likely to stand up for themselves or retaliate, and more likely to accept the derogatory messages sent to them as warranted, which should encourage further victimization.

Moreover, being disliked or not being well-liked puts youth in a vulnerable social position, which could also invite victimization from peers prone to bullying. Peer rejection was found to lead to increases in victimization among elementary-school children (Demol et al., 2020; Hodges & Perry, 1999; Ladd & Troop-Gordon, 2003; Salmivalli & Isaacs, 2005; Sentse et al., 2015; Serdiouk et al., 2015) and adolescents (Sentse et al., 2015; Xiong et al., 2025). Youth generally know which classmates are rejected. Bullying perpetrators likely feel safer targeting less-liked peers as they are less likely to be defended (Laninga-Wijnen, Garandeau, et al., 2025; Sainio et al., 2011) and victimizing them might be perceived more positively by peers. Therefore, perpetrators do not have to fear retaliation from those who like the victim or losses in status from the peer group. This might be especially true in early adolescence, when abstract thinking allows youth to make hypotheses about the possible consequences of their behavior.

## Moderating Role of Classroom Average of Internalizing Symptoms and Peer Status

The extent to which internalizing problems make children more susceptible to peer victimization could depend on how prevalent these symptoms are in the classroom. The person-group dissimilarity model (Wright et al., 1986) postulates that individuals are more likely to be rejected by the group when they deviate from the group norm. Indeed, youth who are more withdrawn were found to be less accepted in classrooms where withdrawal was less common (Chang, 2004, but see Boor-Klip et al., 2017). Thus, the same individual characteristic may be accepted in one classroom but trigger social sanctions (such as victimization) in another. As individuals tend to better like those who are more similar to themselves (similarity-attraction hypothesis; Byrne, 1961), they should be less likely to target peers with aggression because of these traits. Consistent with this theory, cross-sectional research on constructs closely related to internalizing problems found social withdrawal (Bass et al., 2016) to be positively associated with victimization only in classrooms with a low prevalence of withdrawal and scoring higher than the classroom norm in neuroticism to be associated with the highest levels of victimization (Boele et al., 2017). In classrooms where few students experience internalizing symptoms, such problems may be more noticeable and generate less empathy, making these children even more exposed to victimization.

However, the literature is not entirely consistent on this question. Most support for the person-group dissimilarity model comes from cross-sectional research conducted with children rather than adolescents and examining the effects of aggression or withdrawal on peer preference, rather than victimization (e.g., Stormshak et al., 1999). The only longitudinal study testing this model for internalizing problems and victimization found evidence that adolescents who deviated from the classroom norm by being more socially anxious than the classroom average were more likely to increase in self-reported victimization throughout the school year, but no such effect was found for bully-reported victimization (Kaufman et al., 2022). Further empirical tests of the person-group dissimilarity model in relation to psychological difficulties and increases in peer victimization are needed, using different informants for victimization.

Regarding peer status, it would make sense that those who are not accepted by peers would be even more at risk for victimization in classrooms where, on average, students are well-accepted. In classrooms where most are accepted, the lack of peer support that likely accompanies being rejected may be more salient and designate these youth as obvious targets for classmates seeking to harass peers at little cost. However, findings from the only study testing this hypothesis were also inconsistent across informants. Kaufman et al. (2022) found evidence that youth who deviated from their classroom norm by having lower status (operationalized as having fewer friendships and social media connections) than the classroom average were more likely to become victimized according to bully-reports, but not according to self-reports.

## Moderating Role of Classroom Size

Students’ behavior may also be affected by the size of the classroom network. Although findings vary across studies, there is evidence that bullying and victimization levels tend to be higher in smaller classrooms (e.g., Garandeau et al., 2014, 2019; Saarento et al., 2013; Vervoort et al., 2010). One possible explanation is that some perpetrators carry out their actions by manipulating the whole peer group (see Garandeau & Cillessen, 2006) and exerting such influence may be easier in smaller groups. Indeed, bullies were found to have more social power in smaller classrooms (Garandeau et al., 2019). Furthermore, anxious and low-status children were found to be more victimized—according to peer- and self-reports—in smaller classrooms (Saarento et al., 2013).

In line with these findings, we reasoned that vulnerable students might be especially at risk for victimization in smaller classrooms, for several reasons. First, psychological and social problems may be more salient and therefore more easily detected in smaller networks. Second, it might be harder for anxious, depressed and rejected children to find peers who affiliate with them in groups where affiliation opportunities are numerically limited, making their vulnerable position seem even worse and likely to persist. In one of the samples used in Garandeau et al. (2019), the negative association between victimization and popularity was stronger in smaller classrooms, suggesting that low-status youth may indeed be particularly at risk in such classrooms.

## The Present Study

In the field of bullying, it is often assumed that students’ individual characteristics interact with contextual characteristics to determine who is most at risk for victimization. In particular, the well-known person-group dissimilarity model would predict that the main predictors of victimization—internalizing problems and low peer status—would be associated with a lower risk in contexts where their prevalence is higher. However, longitudinal research, which is essential to examine this question—especially since these risk factors are also consequences of victimization—is lacking. The findings of the only study testing this question have been inconsistent depending on the informant of victimization. Specific knowledge of these possible interactions between individual and context are important to guide anti-bullying practices.

This study tests a vulnerability-by-context model by investigating whether internalizing problems and low peer status put early adolescents more at risk for victimization in some classrooms than others. Specifically, we examine whether the classroom average levels of internalizing problems and peer status and the size of the classroom moderate the concurrent and prospective effects of internalizing symptoms and peer status on victimization. First, we expected that higher levels of internalizing problems and lower peer status would be associated with higher victimization, concurrently and 5 months later. Second, we expected that these associations would be stronger in classrooms with lower average levels of internalizing problems and peer status, respectively, and in smaller classrooms, such that adolescents who are highly anxious and depressed and those who have low peer status would be bullied more in such classrooms. Our focus is on how some classroom contexts can exacerbate youth’s preexisting vulnerabilities for victimization, rather than how deviating from the norm puts youth at risk for victimization. Thus, our hypotheses focus on high internalizing problems and low peer status, and we do not have corresponding hypotheses for those low in internalizing problems (or high in peer status) in classrooms with high average levels of internalizing problems (or low average status).

We consider both peer- and self-reported victimization since (a) adjustment correlates of victimization have been shown to differ depending on the informant of victimization (e.g., Bouman et al., 2012), (b) peer-reported victimization, which has the advantage of relying on multiple informants, relies on peers’ awareness of the bullying and therefore may not reflect less visible cases and (c) only self-reported scores capture frequency of victimization, which is better suited to our longitudinal design. Our measure of peer-reported victimization covers the various forms of aggression that victims can be subjected to, which include physical, verbal, and relational aggression as well as social exclusion. As the onset of internalizing psychopathology is especially likely in adolescence (Rapee et al., 2019) and among girls (Durbeej et al., 2019), and victimization tends to decline with age and affect boys slightly more (Smith et al., 2019), our models controlled for gender and age.

## Method

### Participants and Procedure

Data were collected in 18 Finnish secondary schools as part of the STRIVE longitudinal project. We used two waves collected toward the beginning (September 2022, T1) and the middle (January 2023, T2) of one academic year among seventh to ninth graders. All participants received parental consent and gave their assent. At T1, the sample included 4,021 participants (49.6% boys, 46.7% girls, 3.6% other/prefer not to say) from 386 classrooms. The participation rate for T1 was 59.3%, ranging from 6% to 100% across classrooms. At T2, the sample included 381 classrooms with 4,039 participants (49.3% boys, 47.1% girls, 3.6% other/prefer not to say) including 3,584 students who had already participated at T1. The T2 participation rate was 59.7%, ranging from 10% to 100% across classrooms. In Finnish secondary schools, students remain with the same classmates for most of the school day. The students were equally distributed across grades: 35.5% in Grade 7 (*M*_ageT1_ = 12.84; *SD* = 0.52), 33.8% in Grade 8 (*M*_ageT1_ = 13.85; *SD* = 0.53), and 30.7% in Grade 9 (*M*_ageT1_ = 14.81; *SD* = 0.48). All schools were public schools, like >95% of basic education schools in Finland. Each school tends to include students from diverse socioeconomic backgrounds and there is no strong heterogeneity among schools. Students typically go to their neighborhood school and, despite some socioeconomic differences between neighborhoods, the level of segregation is low. The system aims to guarantee an equally good education to all children, regardless of where they live. All schools are required to have a plan for safeguarding students from bullying, violence, and harassment.

To ensure reliability and validity of the peer-nomination scores and classroom-level variables, we selected classrooms with at least 10 participants and a participation rate of at least 40% at both waves, resulting in a sample of 2,641 students (*M*_ageT1_ = 13.71; *SD* = 0.91) from 212 classrooms in 18 schools. The gender distribution was as follows: 48.8% identified as girls, 47.8% as boy, 3.4% as other or preferred not to answer. The average classroom size was 19.8 (range: 13–25).

### Sample and Procedure

The administration of the questionnaires was done online, during regular teaching hours, and was supervised by teachers who had been thoroughly instructed prior to data collection. The participants were assured of the confidentiality of their answers and it was made clear to them that participation was voluntary and they could withdraw at any time. The study was approved by the Ethics Committee for Human Sciences at the University of Turku (decision made on November 18, 2021).

### Measures

#### Internalizing Problems

The 11-item version of the Revised Children’s Anxiety and Depression Scale (RCADS) designed to identify anxiety and depressive disorders in adolescents (Radez et al., 2021) was used to assess internalizing problems. This shorter version, chosen due to time constraints, was shown to have similar sensitivity/specificity values as the RCADS-47-C/P (Radez et al., 2021). Six items captured anxiety (e.g., *I feel anxious when I go to bed at night, I’m afraid of embarrassing myself, I have trouble going to school in the morning because I’m so nervous or scared*). Five items captured depressive symptoms (e.g., *Nothing feels good anymore, I feel worthless, I don’t have the energy to do things*). Answers were given on a 4-point Likert-type scale, ranging from 1 = never to 4 = most of the time. All items were averaged to create a scale for internalizing problems, with a reliability of ω = .92 at T1 (Cronbach’s α was also .92). Confirmatory factor analysis showed good fit to the present data of the one-factor model (CFI = .963, SRMR = .029; RMSEA 90% CI = .071–.082).

#### Peer Status (Rejection/Acceptance)

Peer status was assessed via peer ratings, which have been used in past studies (e.g., Garandeau & Lansu, 2019). Each participant was asked “How much do you like each of your classmates?” and could give a rating (ranging from 1 = *not at all* to 7 = *a lot*) to each other participating classmate. For each student, the received ratings were averaged to create a peer rejection/acceptance score. By allowing participants to indicate the extent to which they like or do not like a classmate, peer ratings provide more precise information than peer nominations (see Cillessen & Marks, 2017), which indicate the proportion of classmates who nominate a student as someone they like the most or someone they like the least.

#### Peer-Reported Victimization at T1 and T2

Four peer-nominated items were used to assess peer victimization via physical aggression, verbal aggression, relational aggression and social exclusion. Each item asked participants who in their class was treated in the following ways: (a) S/He is pushed and hit, (b) S/He is called with nasty names or made fun of, (c) Nasty stories or lies are told about him or her, and (d) S/He is intentionally left out of the group. Nominations were unlimited. The number of received nominations for each item were summed and divided by the number of possible nominators within each class. For each participant, a score was computed by averaging the proportion scores across the four items. The scale had a reliability of ω = .81 at T1 and at T2. Cronbach’s α was .75 at T1 and .81 at T2. Confirmatory factor analysis showed that the fit of the present data to the one-factor model was very good at T1 (CFI = 1.000, SRMR = .001, RMSEA 90% CI = .000–.038) and at T2 (CFI = .999, SRMR = .006, RMSEA 90% CI = .006–.072).

#### Self-Reported Victimization at T1 and T2

The global item from the revised Olweus Bully / Victim Questionnaire (Olweus, 1996), which captures how frequently a student has been bullied, was used to assess self-reported victimization. Each participant was asked “How often has one (or some) of your classmates bullied you in the last couple of months?” The answers were given on the following 5-point Likert-type scale: 1 = *not at all*, 2 = *once or twice*, 3 = *2–3 times a month*, 4 = *once every week*, and 5 = *several times a week*. This global item was shown to have good construct validity and psychometric properties (Solberg & Olweus, 2003).

#### Classroom Characteristics

*Classroom mean levels of internalizing problems* and *classroom mean levels of peer status* were computed by averaging, within each classroom, all individual scores for internalizing problems and peer status, respectively. Classroom size was the total number of students in each classroom, including nonparticipants.

#### Demographic Information

Gender and age were self-reported. Gender was dummy coded as 1 = *boy*, 0 = *girl*, since less than 4% of participants indicated “other/prefer not to answer.” From a list of different ages ranging from 10 to 18 years, participants selected the one corresponding to their age.

### Analytic Plan

Multilevel analyses were conducted in M*plus 8.6* to test whether classroom features moderated the extent to which internalizing problems and peer rejection were associated with concurrent and future peer victimization. The first series of models tested concurrent associations at T1, with separate models for self-reported victimization and peer-reported victimization as outcomes. After testing the unconditional model to obtain the intraclass correlations (ICC), we ran Models 1a and 2a (for peer-reported and self-reported victimization, respectively) including only individual-level predictors (age, gender, internalizing problems, and peer status). Next, we added three classroom-level predictors, which were average levels of internalizing problems, average peer status, and classroom size (Models 1b and 2b). We then tested whether the random slopes (internalizing and victimization, peer status and victimization) significantly varied across classrooms. Finally, to test the possible moderating effect of classroom characteristics, we included cross-level interactions (Models 1c and 2c), via which we tested whether (a) classroom mean levels of internalizing significantly predicted the random slope between internalizing and victimization, (b) classroom mean levels of peer status significantly predicted the random slope between peer status and victimization, and (c) classroom size significantly predicted both random slopes. The second series of models tested the effects of the same T1 predictors on T2 victimization, controlling for T1 victimization. The models are referred to as 3a, 3b, and 3c for peer-reported victimization and 4a, 4b, and 4c for self-reported victimization. Significant interactions will be decomposed using simple slopes at high (+1 *SD*) and low (−1 *SD*) levels of the classroom-level variable and high (+1 *SD*) and low (−1 *SD*) levels of the individual-level predictor.

All individual-level predictors, except age, were centered at the classroom mean. Age and classroom-level variables were centered at the grand mean. We used Bayes estimation. The proportion of missing data was low: 0% for all peer-reported variables, 0.9% and 3% for self-reported victimization at T1 and T2, respectively, 3.5% for T1 internalizing problems, and 4% for gender. Missing values were handled with Bayesian imputation.

## Results

The intra-class correlations (ICC) indicated that 12.2% and 5.7% of the variance in T1 peer-reported and self-reported victimization, respectively, was due to between-classroom differences. At T2, these percentages rose to 21.7% for peer-reported and 6.1% for self-reported victimization. Descriptive statistics and correlations for all variables are shown in Table 1. Results for the concurrent multilevel analyses predicting T1 peer-reported and self-reported victimization are presented in Tables 2 and 3, respectively. Results for the longitudinal multilevel analyses predicting T2 peer-reported and self-reported victimization are presented in Tables 4 and 5, respectively.

**Table 1.** Means (Standard Deviations), Range and Correlations for the Main Study Variables (Uncentered).

	*M* (*SD*)	*N*	Range	1.	2.	3.	4.	5.	6.
Individual-level variables									
1. Age (in years)	13.71 (.91)	2,630	10–18	-					
2. Peer-reported victimization T1	.01 (.04)	2,641	.00–.44	.05*	-				
3. Self-reported victimization T1	1.27 (.78)	2,618	1–5	.06**	.32***	-			
4. Internalizing problems T1	2.12 (.70)	2,549	1– 4	.06**	.08***	.21***	-		
5. Peer status T1	4.17 (.77)	2,640	1.64–6.40	.05**	−.26***	–.12***	.02	-	
6. Peer-reported victimization T2	.02 (.04)	2,641	.00–.54	.02	.63***	.24***	.06**	−.25***	-
7. Self-reported victimization T2	1.37 (.90)	2,561	1–5	.01	.25***	.47***	.19***	−.09***	.29***
Classroom-level variables									
1. Peer-reported victimization T1	.01 (.02)	212	.00–.10	-					
2. Self-reported victimization T1	1.27 (.30)	212	1.00–3.10	.54***	-				
3. Internalizing problems T1	2.13 (.24)	212	1.61–2.78	.23***	.23***	-			
4. Peer status T1	4.18 (.48)	212	3.14–5.68	−.21**	−.14*	.05	-		
5. Size T1	19.77 (2.75)	212	13–25	−.02	.08	.03	−.11	-	
6. Peer-reported victimization T2	.02 (.02)	212	.00–.20	.42***	.28***	.15*	−.12	.00	-
7. Self-reported victimization T2	1.38 (.34)	212	1.00–2.76	.39***	.57***	.21**	−.11	.02	.41***

* *p* < .05, **p < .01, ***p < .001.

**Table 2.** Unstandardized Estimates for Concurrent Models Predicting Peer-Reported Victimization at Time 1.

	Model 1a			Model 1b			Model 1c		
	Est. (*SE*)	95% CI	*p*	Est. (*SE*)	95% CI	*p*	Est. (*SE*)	95% CI	*p*
**Individual-level predictors**									
Age	−.003 (.001)	−.006, −.001	<.001	−.003 (.001)	−.006, −.001	<.001	−.004 (.001)	−.005, −.001	.020
Boy	.008 (.002)	.005, .011	<.001	.008 (.001)	.005, .011	<.001	.009 (.002)	.006, .012	<.001
Internalizing	.005 (.001)	.003, .007	<.001	.006 (.001)	.003, .008	<.001	.005 (.002)	.001, .008	<.001
Peer status	−.015 (.001)	−.017, −.013	<.001	−.015 (.001)	−.017, −.013	<.001	−.015 (.002)	−.018, −.011	<.001
**Classroom-level predictors**									
Mean level of internalizing				.018 (.006)	.006, .030	<.001	.018 (.007)	.006, .031	.020
Mean level of status				−.007 (.003)	−.015, −.002	<.001	−.007 (.002)	−.012, −.003	<.001
Classroom size				.000 (.000)	−.001, .000	.520	.000 (.000)	−.001, .001	.600
**Cross-level interactions**									
Class internalizing × Internalizing							.001 (.007)	−.012, .014	.800
Class status × Status							.005 (.004)	−.004, .013	.160
Class size × Internalizing							.001 (.001)	−.001, .002	.320
Class size × Status							.000 (.001)	−.001, .002	.780

*Note. N* = 2,641.

**Table 3.** Unstandardized Estimates for Concurrent Models Predicting Self-Reported Victimization at Time 1.

	Model 2a			Model 2b			Model 2c		
	Est. (*SE*)	95% CI	*p*	Est. (*SE*)	95% CI	*p*	Est. (*SE*)	95% CI	*p*
**Individual-level predictors**									
Age	.055 (.020)	.008, .092	.020	.050 (.020)	.015, .089	<.001	.045 (.020)	.003, .081	.030
Boy	.169 (.037)	.103, .247	<.001	.172 (.035)	.092, .243	<.001	.171 (.034)	.109, .244	<.001
Internalizing	.295 (.025)	.247, .346	<.001	.299 (.026)	.249, .347	<.001	.300 (.033)	.230, .358	<.001
Peer status	–.139 (.027)	–.193, –.085	<.001	–.137 (.022)	–.181, –.095	<.001	–.135 (.030)	–.203, –.087	<.001
**Classroom-level predictors**									
Mean level of internalizing				.235 (.086)	.006, .030	.010	.226 (.081)	.074, .380	<.001
Mean level of status				–.098 (.038)	–.177, –.032	<.001	–.091 (.041)	–.177, –.032	.036
Classroom size				.007 (.006)	–.006, .019	.210	.005 (.007)	–.009, .019	.380
**Cross-level interactions**									
Class internalizing × Internalizing							–.010 (.141)	–.272, .273	.940
Class status × Status							.005 (.004)	–.026, .023	.830
Class size × Internalizing							.033 (.059)	–.068, .156	.530
Class size × Status							–.016 (.009)	–.033, .002	.086

*Note. N* = 2,641.

**Table 4.** Unstandardized Estimates for Longitudinal Models Predicting Peer-Reported Victimization at Time 2.

	Model 3a			Model 3b			Model 3c		
	Est. (*SE*)	95% CI	*p*	Est. (*SE*)	95% CI	*p*	Est. (*SE*)	95% CI	*p*
**Individual-level predictors**									
Age	.002 (.001)	.000, .004	.060	.002 (.001)	.000, .004	.020	.002 (.001)	.000, .004	.040
Boy	.002 (.001)	.000, .005	.060	.003 (.001)	.000, .005	<.001	.003 (.001)	.001, .006	<.001
Internalizing	.001 (.001)	–.001, .003	.340	.001 (.001)	–.001, .004	.160	.002 (.001)	–.001, .004	.160
Peer status	–.008 (.001)	–.009, –.006	<.001	–.008 (.001)	–.009, –.006	<.001	–.008 (.002)	–.012, –.005	<.001
Victimization T1	.750 (.017)	.716, .792	<.001	.750 (.019)	.711, .786	<.001	.754 (.018)	.722, .798	<.001
**Classroom-level predictors**									
Mean level of internalizing				.014 (.007)	.006, .030	.040	.016 (.008)	.002, .031	.020
Mean level of status				–.007 (.004)	–.020, –.014	.040	–.007 (.004)	–.014, .000	.040
Classroom size				.000 (.001)	–.002, .001	.780	.000 (.001)	–.001, .001	.760
**Cross-level interactions**									
Class internalizing × Internalizing							–.010 (.006)	–.021, .001	.060
Class status × Status							.003 (.004)	–.004, .010	.420
Class size × Internalizing							.000 (.001)	–.001, .001	.940
Class size × Status							.000 (.001)	–.001, .002	.600

*Note. N* = 2,641.

**Table 5.** Unstandardized Estimates for Longitudinal Models Predicting Self-Reported Victimization at Time 2.

	Model 4a			Model 4b			Model 4c		
	Est. (*SE*)	95% CI	*p*	Est. (*SE*)	95% CI	*p*	Est. (*SE*)	95% CI	*p*
**Individual-level predictors**									
Age	.022 (.021)	–.021, .062	.300	.012 (.021)	–.027, .053	.560	.010 (.027)	–.044, .061	.670
Boy	.003 (.039)	–.070, .072	.980	.003 (.039)	–.078, .080	.940	–.004 (.034)	–.071, .061	.940
Internalizing	.127 (.024)	.084, .174	<.001	.131 (.028)	.067, .181	<.001	.116 (.033)	.038, .168	.010
Peer status	–.056 (.026)	–.102, –.004	.020	–.054 (.023)	–.099, –.013	<.001	–.063 (.027)	–.111, –.002	.040
Victimization T1	.486 (.023)	.441, .527	<.001	.489 (.022)	.442, .520	<.001	.484 (.022)	.437, .530	<.001
**Classroom-level predictors**									
Mean level of internalizing				.236 (.098)	.047, .448	<.001	.235 (.103)	.032, .432	.010
Mean level of status				–.081 (.046)	–.168, .021	.100	–.073 (.046)	–.168, .014	.140
Classroom size				–.001(.008)	–.016, .017	.920	–.001(.009)	–.022, .014	.920
**Cross-level interactions**									
Class internalizing × Internalizing							.052 (.126)	–.204, .259	.650
Class status × Status							.010 (.065)	–.095, .146	.940
Class size × Internalizing							–.003 (.010)	–.020, .016	.720
Class size × Status							.005 (.009)	–.014, .024	.560

*Note. N* = 2,618.

### Main Effects of Internalizing Symptoms and Low Peer Status on Victimization

#### Concurrent Analyses

The main effects of T1 individual-level predictors on T1 victimization were tested in Models 1a and 2a (see Tables 2 and 3 for peer-reported and self-reported victimization). These predictors explained around 10% and 8% of the within-classroom variance in peer-reported and self-reported victimization, respectively. Having higher levels of internalizing problems, lower status and identifying as a boy were associated with both indicators of victimization. Older students were less likely to be nominated as victims by their classmates, but more likely to report being victimized.

The results of Models 1b and 2b show that levels of victimization were higher in classrooms with higher average levels of internalizing problems and in classrooms where, on average, students received lower status ratings. There was no significant effect of classroom size. These classroom-level predictors explained around 17% and 6% of the between-classroom variance in peer-reported and self-reported victimization, respectively.

#### Longitudinal Analyses

The main effects of T1 individual-level predictors on T2 victimization, controlling for T1 victimization, were tested in Model 3a and 4a (see Tables 4 and 5, for peer-reported and self-reported victimization, respectively). These individual-level predictors explained around 49% and 21% of the within-classroom variance in T2 peer-reported and self-reported victimization, respectively. Once T1 peer-reported victimization was controlled for, low peer status emerged as a significant predictor of both indicators of victimization. Higher levels of internalizing problems were associated with T2 self-reported victimization, but not with T2 peer-reported victimization. For both indicators of victimization, T1 victimization was strongly positively associated with T2 victimization, and the effects of age and gender were not significant.

The main effects of the classroom-level predictors on T2 victimization are shown in Models 3b and 4b. These predictors explained around 2% and 8% of the between-classroom variance in T2 peer-reported and self-reported victimization, respectively. Levels of T2 victimization were higher in classrooms where students, on average, experienced more internalizing problems. Higher classroom average status at T1 predicted lower T2 peer-reported but not T2 self-reported victimization. Classroom size had no significant effect on T2 victimization, regardless of the indicator.

### Moderating Effects of Classroom-Levels Factors

#### Concurrent Analyses

To examine whether the associations of T1 individual-level internalizing symptoms and status with T1 victimization significantly varied across classrooms, we built a model (not shown in Tables) to estimate the variance of the random slopes for internalizing and status, separately for peer-reported and self-reported victimization. Both variances were significant for peer-reported victimization (*Var* = .0004, *SE* = .00004, *p* < .001 for internalizing; *Var* = .0006, *SE* = .00008, *p* < .001 for status) and for self-reported victimization (*Var* = .094, *SE* = .020, *p* < .001 for internalizing; *Var* = .047, *SE* = .013, *p* < .001 for status). Since these associations significantly varied across classrooms, we added cross-level interactions in Models 1c and 2c to examine whether this variation was explained by the classroom prevalence of the risk factor and by classroom size (see Tables 2 and 3). However, none of these interactions were significant, suggesting that the concurrent effect of internalizing problems on victimization did not vary as a function of the average level of internalizing problems in the classroom, nor classroom size. Similarly, the concurrent effect of status on victimization did not vary as a function of the mean status level of the students in the classroom, nor classroom size.

#### Longitudinal Analyses

As with concurrent analyses, we first examined whether the associations of T1 individual-level internalizing symptoms and status with T2 victimization significantly varied across classrooms by building models (not shown in Tables) to estimate the variance of the random slopes for internalizing and status, separately for T2 peer-reported and self-reported victimization. The variance of the random slopes of interest were significant for both peer-reported victimization (*Var* = .0005, *SE* = .00005, *p* < .001 for internalizing; *Var* = .0007, *SE* = .00008, *p* < .001 for status) and self-reported victimization (*Var* = .061, *SE* = .018, *p* < .001 for internalizing; *Var* = .062, *SE* = .021, *p* < .001 for status). We therefore added cross-level interactions in Models 3c and 4c to examine whether this variation was explained by the classroom prevalence of the risk factor and by classroom size (see Tables 4 and 5). None of the cross-level interactions were significant, suggesting that the between-classroom variance was not explained by any of the three classroom-level predictors included in our analyses.

## Discussion

In recent decades, research on school bullying has brought to light the importance of the classroom social environment (Salmivalli, 2010). Not only can it explain differences in the prevalence of victimization (Pouwels & Garandeau, 2021; Saarento et al., 2015), but some classroom contexts can exacerbate the consequences of being bullied on psychological adjustment (Garandeau et al., 2018; Laninga-Wijnen, Yanagida, et al., 2025). Guided by these findings, this study tested a vulnerability-by-context model by investigating whether the effects of well-known risk factors for peer victimization, namely internalizing problems and low peer status, put youth even more at risk in some classroom contexts than others. Based on theory—in particular, the person-group dissimilarity model—and on prior findings, we focused on two classroom features: Classroom mean levels of the risk factor and classroom size. As longitudinal studies examining these effects are scarce, we used both concurrent and longitudinal designs. Moreover, our analyses included two operationalizations of victimization: Peer-reported victimization, which indicates the extent to which a student has a reputation for being bullied, and self-reported victimization, which is a measure of how frequently a student feels victimized by others.

Our first goal was to examine whether internalizing problems and low peer status were concurrently and prospectively associated with higher victimization. In line with the literature, our results showed that, concurrently, adolescents experiencing more internalizing problems (e.g., Laninga-Wijnen, Yanagida, et al., 2025) and adolescents with lower status (e.g., Kärnä et al., 2010) were more victimized, both according to peer-reports and self-reports. Longitudinally, internalizing predicted higher self-reported victimization, which is consistent with prior research (e.g., Christina et al., 2021), but surprisingly it did not predict peer-reported victimization. This might be partly due to shared method bias but could also reflect the fact that those who report feeling anxious and depressed might be more prone to report being victimized even when the peer group does not recognize this victimization. Our prospective findings also show that adolescents with low peer status were more likely to report being victimized or to be seen as a victim by classmates 5 months later. Among the studies examining this prospective association, few were conducted with adolescents, and found support for such link (e.g., Sentse et al., 2015).

With regard to the role of the classroom context, we had anticipated that, in line with the person-group dissimilarity model (Wright et al., 1986), being anxious and depressed would be less likely to lead to victimization in contexts where these characteristics are more prevalent. Presumably, these characteristics stand out more and generate less empathy in contexts where they are uncommon. However, no support was found for a vulnerability-by-context model concurrently or longitudinally for the classroom-level moderators we considered, and regardless of the informant for victimization. Caution is warranted in the interpretation of these nonsignificant interaction effects, as they may be due either to the actual absence of moderation of these classroom characteristics but could also be influenced by inadequate variation in the predictor or moderator (see McClelland & Judd, 1993). Indeed, few students scored extremely high on internalizing problems.

These results are not fully consistent with the findings of Kaufmann et al. (2022) that being dissimilar from the classroom norm in terms of being more anxious predicted higher self-reported victimization (though not bully-reported victimization) and being dissimilar from the classroom norm in terms of having fewer friends predicted higher bully-reported victimization (though not self-reported victimization). It should be noted however that peer rejection (i.e., being less liked or disliked), which was the focus of our study is distinct from number of friends or social connections.

As our analyses still showed that there was significant variation between classrooms in the extent to which these individual characteristics led to victimization, we cannot interpret the present findings as an indication that the robust associations of internalizing problems and low peer status with peer victimization are not context-sensitive. A more likely explanation is that other classroom features explain this variation. Although the cross-sectional study by Saarento et al. (2013) suggested that the size of the classroom peer network mattered for the anxiety-victimization and rejection-victimization associations, our findings did not support such moderating effects of classroom size for either individual risk factor. However, there is some indication in the literature that the distribution of power in the classroom—or status hierarchy—might play a role. Not only does status hierarchy tend to promote bullying (e.g., Garandeau et al., 2014; Malamut et al., 2024), but shyness was found to be positively associated with peer victimization only in high-hierarchy classrooms, that is, in classrooms with stronger inequalities in peer status (Hu et al., 2023). Similarly, disliked youth were found to be more likely to increase in victimization in classrooms that were more hierarchical in the sense of “centralization of victimization” meaning that most students are not victimized and bullying is targeted at only a few (Serdiouk et al., 2015).

It is also possible that any classroom-level factors that encourage bullying behavior put at risk first and foremost those who are psychologically and socially vulnerable. Such classroom-level factors include rates of certain bystander responses in bullying situations. Indeed, levels of bullying were found to be higher in classrooms with higher levels of reinforcing of the bullying behavior (Salmivalli et al., 2011; Thornberg & Wänström, 2018) and lower levels of defending victims (Salmivalli et al., 2011), and cross-sectional studies have shown that the associations of psychological or social maladjustment with victimization were stronger in classrooms that were higher in bullying-reinforcing and lower in victim-defending (e.g., Kärnä et al., 2010). Any context where aggression is condoned or viewed positively, such as classrooms with high levels of moral disengagement (Thornberg et al., 2023) or where teachers do not use disciplinary actions against bullying (Burger et al., 2022) or are not authoritative enough (Kloo et al., 2023), is associated with higher levels of victimization or bullying, and it is likely that the targets of this aggression are the students who appear the most vulnerable.

The lack of significant moderating effect of the classroom prevalence of internalizing and peer status may also be related to measurement. The classroom was chosen as the unit of analysis, but the various peer contexts adolescents belong to range from the close-knit group of friends to the grade-level peer network. The moderating effect of contextual norms may differ across these different group levels. Indeed, in a recent study, Bass et al. (2022) sought to further understand the person-group dissimilarity effect by examining whether violating group norms was more likely to be sanctioned with victimization when the norms were proximal (i.e., those of a small clique of less than 10 students) versus distal (e.g., school-wide). They found that adolescent peer groups enforced proximal group norms more than distal norms. Following this reasoning, the classroom may be divided into distinct peer groups and the normativeness of internalizing and peer rejection within these peer groups, for example girls’ groups and boys’ groups, may matter more for the likelihood of becoming victimized than the classroom average. Indeed, classroom norms may be more likely to moderate links between rejection and victimization when computed and tested separately for each gender (Isaacs et al., 2013). This illustrates the disadvantages of aggregating individual scores at the classroom level. There might also be heterogeneity among classrooms with the same mean. A classroom with a mean close to the sample mean might include students who all have the same medium level of the trait, or it could include both students scoring extremely high and students scoring extremely low on that trait.

## Conclusion

This study replicated the concurrent findings that early adolescents who are victimized tend to experience more internalizing problems and to be less liked than their nonvictimized counterparts. Longitudinally, it replicated the finding that having low status (being disliked) puts youth at risk of being victimized over time. Being more anxious and depressed emerged as a prospective risk factor only for self-reported victimization. Taken together, these findings suggest that bullying-prevention efforts should focus on the social integration of rejected children even if they are not being victimized and on reducing internalizing problems as part of universal school-based programs. Either concurrently or longitudinally, the links between these individual vulnerabilities—internalizing problems and low status—and victimization did not depend on how prevalent this characteristic was in the classroom, nor on the size of the classroom. These associations did differ across classrooms, however, suggesting that other features of the classroom social context must explain this variation.

## Notes

1. Peer rejection (and peer acceptance) refer to being disliked (and being liked) and are typically measured by asking participants to nominate the classmates they like the least (and the most), and dividing, for each student, the sum of received nominations by the number of nominators. In this study, acceptance and rejection were assessed with a single measure using peer ratings. Each participant evaluated the extent to which they liked each classmate from 0 (*not at all*) to 7 (*a lot*) and the acceptance/rejection score—or peer status score—was computed by averaging the received ratings for each student.
